# Observations on Apoplexy, Illustrated by Cases

**Published:** 1803-04-01

**Authors:** J. Magennis


### Observations *on* Apoplexy, *illustrated by* Cases; *by* J. Magennis, *M. D.*

THERE has been already so much said on Apoplexy, and so many sheets of your valuable Journal occupied by the Controversy to which it has given rise, that there seems some degree of hardihood in venturing to renew the subject; but to acquire just notions of this formidable malady is so important to mankind, that any Observations that have a tendency to elucidate a point in which so many have differed, will, I trust, suffice to plead an apology for bringing before the public once more, a question which, by a kind of tacit consent, seems to have been abandoned by both parties, without the arguments adduced, I fear, bringing conviction to either.

My attention has been awakened to the consideration of the subject by a case, detailed in the sequel, which lately occurred at the Royal Hospital, and by the painful Controversy above alluded to. Comparing past experience with more recent observation and reflection, there results from the comparison a persuasion that Apoplexy arises much oftener from congestion, or a preternatural accumulation of blood in the vessels of the head, than from extravasation or exudation. This opinion is strengthened not only by the history of the following Cases, and many others related by men of the first eminence in the profession, but also by the frequent and rapid recoveries from such attacks. Dr. Quin, of Dublin, who has written an excellent Treatise on Apoplexia Hydrocephalica, cites two Cases, where no extravasation of blood or effusion of lymph lymph was found, a circumstance that excited much surprize in him.

The opportunities afforded of examining into the cause and morbid appearances of patients dying of Apoplexy are few, because this disease generally attacks people in the higher circles of life; the opulent and sedentary, and those capable of indulging in the pleasures and sensualities of a luxuriant table: hence the difficulty of ascertaining its real seat; and hence the prevalence of opinion, that rupture and extravasation are commonly the cause of Apoplexy. But it appears to me, that the vessels of the brain are too closely and firmly supported by the bones of the cranium and surrounding membranes, the dura and pia mater, to be often subject to injuries of this nature, except from great and unusual violence; and I am inclined to think, that a rupture of these vessels, and extravasation of their contained fluids by the mere impetus of the circulation, derived from the force of the heart and arteries, is a circumstance which very rarely occurs.

The speedy and complete relief so frequently experienced in this frightful complaint from blood-letting, both general and local, joined to other evacuations, would seem still farther to strengthen and confirm this supposition; besides, hitherto, the discovery of a series of lymphatics in the brain, has eluded the researches of the most accurate and keen-sighted Anatomists, assisted by all the improvements of modern science. It is indeed to be presumed from analogical reasoning, that Nature, so wise and provident in all her provisions, has not omitted to furnish so essential an organ with an absorbent system, though too minute to be detected by human ingenuity. If there be such an order of vessels, they open principally by very minute, imperceptible points into the ventricles and other cavities of the brain. Admitting, then, the existence of lymphatic veins in this viscus, their extreme minuteness would render them unequal to the office of absorbing such a portion of extravasated fluid as should induce a suspension or temporary abolition of the powers of sense and motion, to account for the speedy and almost immediate relief often derived from a copious bleeding. If it be asserted that the inhalent veins carry on the important function of absorption, it may be answered, that we have no evidence of the red veins supplying the place of lymphatic vessels in other parts of the body, except in the corpora cavernosa penis; and though we admit the existence of those veins, it is probable that they will be rendered less less capable or wholly unfit for performing this office, by the fluids, emancipated from their natural channels, now acting the part of a foreign body in compressing them. It is pretty well ascertained, that in all cases of extravasation occurring from blows or other external injuries received on the head, the patient is only to be relieved by an operation, in order to give issue to the compressing fluid.

It will follow, then, from a due consideration of these premises, that extravasation and exudation, in any given quantity so as to induce a sudden and total abolition of sensation and motion, will commonly, if not invariably, prove fatal. The natural inference deducible from these supposed facts, is, that in almost all recoveries which follow apoplectic attacks, compression of the medullary substance of the nerves from repletion and congestion in the veins and arteries of the brain has been the efficient cause. As dissection has, however, clearly demonstrated the existence of effusions in subjects who have died of this disease, may not these effects have arisen from the mouths of the exhalent arteries being so disposed, just before or immediately after death, from previous repletion and its consequent relaxation, as to permit a portion of blood and serum to pass out, or even to exude through the coats of the vessels themselves? So that what we consider as the cause of dissolution, may only be the consequence of it. And although the vessels of the head should have yielded to the pressure of their distending fluids, this does not necessarily depend on vascular tone and increased rapidity of circulation, but probably on atony and diminished excitement in these vessels favoring a preternatural accumulation.

Without attending particularly to the nosological definition of Apoplexy as usually given by systematic writers, or meaning to include all its varieties, I shall chiefly confine myself to that division of it, characterized under the titles Sanguineous and Serous; which, I think, may be more appropriately defined, a congestion of blood in the vessels of the head inducing a compression of the brain followed by a loss of sense and motion. Of this, there are three principal species; the first arising from increased vascular tone throughout the system, with an undue determination of a rapid circulation to the head, and may be denominated, General Sthenic Congestion. The second arises from local inflammation some how or other induced, that is, inflammation confined almost solely to the head, with little little or no general inflammatory diathesis; this may be defined, Local Sthenic Congestion. The third species is supposed to be derived from an atony or debility pervading the substance of the brainular vessels, thereby rendering them incapable of transmitting the quantity of blood received, which gradually accumulating and distending their coats, ultimately produces compression; this may be aptly enough characterised, Atonic Congestion.

If these definitions be consonant to the phenomena known commonly to attend the different varieties of this fatal disease, above alluded to, when the morbid appearances previous to and after death are carefully observed and compared, what are the rational indications of cure which this classification points out? And is the proper mode of treatment more obvious from Apoplexy thus defined, than under the former titles, which were so denominated from effects observed probably after death, viz. extravasation or effusion of blood and serum, but which have a manifest tendency to mislead the judgment of the practitioner into dangerous errors?

Numerous examples of the first kind are cited by almost every writer, ancient and modern, who has written on this formidable disorder, though probably it is the least frequent of the three. It usually attacks the young and vigorous, at or about the age of puberty, who live high and take violent exercise. It is produced by a variety of causes, and is commonly supposed to terminate in rupture of the vessels and extravasation of blood, or in effusion of coagulable lymph on the meninges or into the ventricles of the brain.

The proper treatment of this species is very obvious; the lancet is to be freely employed, as the system will bear general bleeding to great extent; and here indeed it becomes absolutely necessary to prevent the disease from running into its last and most fatal stage; copious evacuations by the intestines are likewise to be procured; and the application of cold to the head, frequently renewed, will powerfully contribute to calm the internal tumult. These, joined to other subordinate remedies which readily suggest themselves to the experienced practitioner, are the means most likely to succeed in warding off the destructive effects of General Inflammatory Congestion.

James Thomson, a young man twenty-four years of age, very muscular and athletic, had been much exposed for two days to the effects of a burning sun, during which time he laboured hard, and drank freely of spirit and water, water, but not to excess; was three days after suddenly seized with symptoms of compressed brain, on the 3d of May, 1790. He had complained some hours before of severe head-ach and nausea, with lancinating pains through the temples and down the neck. When called to him, which was in twenty minutes after the abolition of sense, and in great measure of motion, I found the countenance of a dusky red, the vessels of the conjunctiva much inflamed, with stertorous breathing and a strong bounding pulse. From twelve to fourteen ounces of blood were immediately taken from the arm; scarifications made on the neck and temples; and as soon as the hair could be removed, cloths dipped in vinegar and water were applied all over the head, and frequently renewed. In a few minutes after these applications he opened his eyes, as if awakened from a profound sleep, and gradually recovered his intellectual powers. In ten days more he was free from complaint. The good effects of the topical cold affusions were here conspicuous; and they will be found, I believe, among the most powerful means of subduing the internal tumult of the brain in similar cases.

Miss W. a young lady about sixteen years of age, who, from a state of the utmost languor and debility owing to chlorosis, had been in the course of three weeks restored to vigorous health by the powerful assistance of steel; but who having continued the use of the medicine too long, and a costive state of the body supervening for several days, was suddenly and unexpectedly attacked with Apoplexy. The medical gentleman under whose care she had been, a man of great abilities and much practical observation, being then in the country, I was sent for, and by my desire Sir F. Milman; but he being at the moment absent on professional duty, did not arrive till long after. I found the patient deprived of all sense and motion, the countenance of a purplish hue, the eyes suffused, attended with stertorous breathing and a hard full pulse. Eight ounces of blood were immediately taken from the arm, an acrid cathartic enema administered, which copiously evacuated the intestines; cupping glasses were applied to the neck and shoulders, by which means seven or eight ounces more of blood were abstracted. Shortly after this she began to recover, gradually regaining her understanding; and by the time Sir F. arrived, she was perfectly collected. There could be no doubt respecting the cause of this attack; she had been for some days previous, in the highest health and spirits, strongly indicated by ruddiness of countenance and firmness firmness of fibre. Thus circumstanced, and still under the powerful influence of the same stimulus which had produced such salutary changes, we cannot be surprized at the consequences that followed. Some weeks elapsed, however, before the system recovered from the bad effects of this excess of stimulation.

The second or local Sthenic Congestion is, I am persuaded, one of the most frequent causes of Apoplexy, and if not arrested by a speedy and judicious treatment, applied early in its commencement, the disease will run rapidly into indirect debility, the elasticity and irritability of the vessels will be diminished, their fibres elongated and weakened, their force and density as living solids gradually impaired and ultimately destroyed; hence accumulation and incurable Congestion.

The modus medendi in this species, is not so obvious as in the former; general bleeding must be employed with greater caution, as it tends much to weaken the vis vitæ, without abating in any considerable degree the morbidly increased action of a part. One general bleeding is therefore admissible and necessary; but it ought to be, in every possible case, from the jugular veins; and if during the operation, compression can be made on the carotid arteries, to retard the circulation to the head, whilst the surcharged sinuses and vessels of the brain are emptying by the emission of blood from the jugulars, the solution of the inflammation will be more speedy and complete. After this, recourse must be had to local blood-letting by leeches to the temples and forehead, as well as cupping and scarifications on the neck; the head shaved, and covered with cloths dipped in cold vinegar frequently renewed; blisters applied along the course of the sutures, will tend to lessen the tension of the communicating vessels by their stimulus and the discharge of serum which they procure from the neighbouring parts.

Both sexes are subject to affections of this nature, those especially who indulge much in the luxuries of the table, and who lead a sedentary and indolent life. It generally attacks persons from the age of forty-five to sixty, who are frequently seized two or three hours after dinner, when the stomach is distended with food, which pressing on the descending trunk of the aorta, determines a larger portion of blood to the carotid and vertebral arteries, while its stimulus increasing the activity of the chyliferous and lymphatic system, supplies the circulation with fresh fuel. The impetus of an additional quantity of blood acting on the vessels of the head, already predisposed probably to inflammation from venal plethora often subsisting at this period of life, powerfully assists in producing local Sthenic Congestion. Great bodily efforts, watchings, fatigue, anxiety, and mental exertions, strongly predispose the habit to this species of Apoplexy.

A gentleman of the first respectability and connexions in life, aged about forty-five, of a fair and florid complexion, and apparently of strong and vigorous health, under considerable anxiety of mind, travelled three or four days and nights without any repose, except what he casually took in the carriage during the journey, was seized a few days afterwards without any previous existing complaint, and after all cause of anxiety had ceased, with symptoms of compressed brain. He had occasion to go out in the morning to transact some business, and with some difficulty found his way home; a few minutes after his return, he complained of pain in the head, and almost instantly lost the power of speech, which may be said he never after regained. The family surgeon was immediately sent for, but unfortunately he was out of the way; a physician of the first eminence was then called in, who did not arrive till between six and seven hours from the commencement of the attack; he directed a very copious bleeding instantly, and made every other possible effort to save the patient, but all to no purpose; he gave at one time some hopes of amendment for a day or two; but like Major, a case to be mentioned, he expired in a few days. The state of the brain was carefully examined after death, and all its vessels were found prodigiously distended with blood, without the least appearance of extravasation or any kind of effusion whatever. Thus Congestion of a few hours standing only, and notwithstanding the ablest assistance and most skilful treatment, terminated fatally in the course of a few days.

How much is it to be lamented that intemperate language and angry invective, are so frequently substituted in medical disputation for dispassionate reasoning and urbanity of manners; in no other professional writers do we observe such heat and illiberality to their opponents, if the reformers and religious controversialists of the sixteenth century be excepted. Much abusive contention has lately arisen respecting the propriety and utility of exhibiting emetics in cases of Apoplexy; and however we may deplore the animosity to which it has given birth, it must be confessed, that this collision of sentiment and contrariety of of opinion, have been the means of eliciting a fund of useful information and much desultory knowledge.

Without meaning to take any side of the question as far as it regards persons, or entering into a justification of either party, it may be asserted with some degree of confidence, that on fair investigation, the truth will be found to lie between both extremes. Whoever attentively considers the mischief that often results from distension of the stomach and bowels overloaded with aliments, will, I am sure, be convinced of the utility and even necessity of sometimes administering an emetic in certain states of Apoplexy. Vomiting may be employed with great benefit and propriety where the attack is known to follow shortly after full eating; but in order to exhibit the remedy with the least possible risk, and at the same time with the greatest possible advantage, it should invariably be preceded by a free emission of blood; this precaution will obviate the apprehended danger of rupturing the vessels or increasing the Congestion by impelling the blood in too great quantity and with too much velocity to the head. It is however to be doubted, whether vomiting is attended with these consequences, notwithstanding the opinion is sanctioned by some of our best medical writers. That the jugular veins are somewhat compressed, and the returning blood momentarily retarded during the short period of ejection, must be acknowledged; yet, when it is considered that these effects last only for a moment, and how powerfully the lymphatics and lacteals of the primæ viæ are emulged during the operation, pouring back their contents into the duodenum and stomach, thereby cutting off all farther supply to the circulation, and consequently preventing as well as diminishing plethora; we shall not hesitate, in certain cases and under certain limitations, to avail ourselves of the advantages that are likely to accrue from the application of so powerful a remedy.

It often requires much accuracy of judgment and nicety of discrimination, joined to great experience, to be always able to distinguish those cases of Apoplexy in which general bleeding would be preferable to local. When the vigor of the constitution is impaired by a long course of intemperance, or the vital energy on the decline from arthritic affections and other diseases, the utmost circumspection is necessary in using the lancet. If however, on mature consideration of all the circumstances, general bleeding be deemed proper, it should at first be done sparingly, and as near the part affected as possible, viz. from the jugular veins, which will speedily and effectually remove the Congestion. But as great debility and exhaustion often unexpectedly follow this operation, topical blood-letting should in general have the preference, as it may be carried to great extent without eventually injuring or weakening the system.

D. G——, Esq. a representative in parliament for one of the northern counties during the eventful period of Lord North's administration, was suddenly seized with an apoplectic fit at a friend's house. This gentleman was sixty years and upwards at the time, rather of a spare habit of body, and for many years of his life subject to gouty affections, but then apparently in good health. He dined at home as usual, and drank near a bottle of claret, his ordinary quantity when in health; walked out late in the evening, and shortly after his arrival was seized. When I saw him, which was about three hours from his being attacked, I found him labouring under every symptom that usually denotes compressed brain; a total abolition of sense and motion had taken place, with deep sleep, laborious and stertorous breathing, a redness and fulness of the face, suffused eyes, accompanied by a strong, full pulse. No case could be more strongly indicative of general bleeding; accordingly, a vein was opened, and ten ounces of blood taken away, the blood bounding in the most violent manner, as if an artery had been opened. I kept my finger on the pulse all the time, and the moment I perceived its vigor abate, the orifice was closed, well aware from his former habits, that he would not bear very copious evacuations, however the urgency of the case might seem to warrant them. But although my patient appeared at first somewhat relieved, yet the energy of the system began gradually to sink from that moment, and to my surprize and mortification, he expired in less than five hours after the bleeding.

I am apprehensive that no treatment, however judicious, would have availed much at the period in question. It may well be supposed that I was extremely anxious to inspect the state of the brain; but unfortunately his lady and friends would not consent to the examination; and I was obliged to rest satisfied with conjecture as to the precise seat and nature of the compression.

The Atonic or Asthenic Congestion, is generally formed by slow and imperceptible gradations, while the body labours probably under a debility from a defect of sensorial power; there is reason to suspect that it is commonly preceded ceded by a species of chronic inflammation of the vessels of the brain, often subsisting for a length of time without exciting any material general tumult in the system, or apprehension in the mind of the patient: slight and wandering pains in different parts of the head of no long continuance, but constantly recurring and chiefly confined to the forehead and temples, sometimes attended with nausea and a contracted brow, mark the insidious approaches of the disease. By degrees other symptoms supervene, and the pains become more fixed, till at last they are concentrated as it were, in the inferior part of the os frontis, between or a little above the superciliary arches, with occasional shootings to the temples, and sometimes in the direction of the longitudinal sinus; the pulse now acquires some farther degree of acceleration, followed by irregular shiverings, giddiness, and all at once the patient tumbles down; he recovers however speedily, rubs his eyes, and is astonished how to account for the fall. This gives the first alarm, and if proper means be not adopted to subdue the complaint, the vessels yielding to repeated pressure and distension, lose their elasticity and contractile powers, are no longer able to propel the quantity of blood received, the accumulation which had been all along gradually forming arrives at its acmé, the congestion becomes complete, abolition of sense and motion follows, and the unfortunate patient, if not relieved, soon closes his career in death.

William Major, a marine belonging to the Plymouth divison, a young man between twenty-one and twenty-two years of age, was received into the Royal Hospital on January 11, 1803. He complained of wandering pains in different parts of the head, chiefly in the forehead and temples, pain about the scrob. cordis, dyspnœa, attended now and then with short irritating cough, but not frequent or troublesome; the pulse was neither hard, full, nor scarcely accelerated beyond the natural standard of health, nor was the tongue in the least degree discoloured; but there was a knitting or contraction of the eye-brows and muscles of the face, which at the time excited no particular attention, nor gave the slightest suspicion of the latent mischief which afterwards discovered itself so fatally; he had upon the whole, to an observer, so few and such slightly marked symptoms of disease, that I felt inclined to believe his complaints feigned; no uncommon occurrence among this description of people; and Mr. Magin, Surgeon to the Marine Infirmary, to whom he had applied some days before his admission, considered it in the same

 light light. The man declared that he had been unwell many days, though he had done duty till the period of his applying to the Infirmary; he had been only discharged from the hospital about seven weeks, having laboured under acute rheumatism, for which it was found necessary to bleed him twice. His appearance now, was altogether the reverse of every thing like subsisting plethora or any tendency to inflammation, the body being thin and seemingly much emaciated, a sallow cadaverous countenance, the face covered with many deeply pitted variolous marks of a coppery hue, having more the resemblance of impressions resulting from the secondary symptoms of an highly aggravated syphilitic taint, the whole interspersed with livid coloured papulæ of a tubercular nature, abounding most on the cheeks and chin.

Such was the general outline of his state; but all this unfavourable apppearance was not in consequence of his present illness, his external condition was nearly the same when under my care before. He likewise complained of nausea and a disposition to diarrhœa; for this a scruple of pulv. ipec. was ordered, to be assisted in its operation by an infusion of flor cham. and two hours after the following mixture: ℞. Ac. am. ac. ℥iss. mist. camp. ℥4or. spt. æther. nitr. ʒiij. syr. pap. alb. ʒiij. M. sumat cochlear. iij quartis horis. On the 12th he felt better, but the following bolus was prescribed to cleanse the intestines, consisting of five grains of calomel and fifteen of rhubarb, and the mixture continued; the pulse regular, the tongue clean and moist; oppression about the præcordia, the cough troublesome. 13th. The general symptoms nearly the same as yesterday, except that he complained of pain in the inferior part of the os frontis, between and above the superciliary arches, to which a small blister was applied. 14th. The dyspnœa and cough more troublesome; a pectoral mixture ordered for the cough, and a blister to the scrob. cordis. On the 15th the affection of the head, which heretofore attracted little attention, now assumed a more threatening and alarming appearance; the vessels of the conjunctiva became turgid and inflamed, the light painful, the pulse accelerated, attended by thirst, anxiety, and darting pains through the forehead and temples. Several ounces of blood were immediately taken away from the arm, and leeches applied to the forehead and temples, which bled copiously. Four hours after these operations, the pains became violent about the temples and between the superior parts of the orbits; in a few minutes he became furious, which state lasted a quarter of an hour, and then suddenly lost the powers of speech, speech, which he never afterwards regained. From this period to the 18th, it was found impossible to convey either medicine or drink into the stomach. In the mean time, purgative enemas were injected, which evacuated the intestines freely; the head was shaved, blisters applied to the sutures, and leeches to the os frontis, above the orbits, as well as again to the temples. During the 16th and 17th, he had a kind of convulsive paroxysm or exacerbation every evening at six o'clock. On the 18th, at the same hour, he was seized with sickness, and vomited a large quantity of greenish bile, after which a profuse perspiration broke out all over the body, and continued for some hours with evident alleviation and remission of former symptoms; for, at this time, the powers of suspended intellect seemed in some measure restored; as he would take whatever was offered, whether medicine or drink, on being desired so to do; but he was totally unable to give utterance to sensation and perception.

After the bleedings and other evacuations, the vigour of the pulse and energy of the system sunk considerably, the adnata recovered its usual transparency, and from the previous state of general emaciation, these active curative means could not, it appeared to me, be pushed farther with any prospect of advantage, although I had not then a doubt respecting the nature of the disease. That the sensorium laboured under compression from Congestion, or effusion of blood or serum, was manifest; and had these evacuations taken place in the very commencement, there is reason to suppose they might have been successful: But the disease made its approaches so insidiously and covertly on a subject apparently so little predisposed to plethora or inflammation, that all suspicion respecting its real nature and tendency was lulled to rest; and when Compression became evident, I was inclined to impute it rather to an accumulation of aqueous fluid in the ventricles than to a preternatural distension of the vessels from red blood, although a principal characterizing symptom of Compression depending on serous effusion was wanting, viz. dilatation of the pupils. On the 19th, the circulation was so languid, with other indications of great exhaustion, that wine and cordial medicines were deemed necessary; but notwithstanding these and other means, he gradually sunk, the suffusion and muddy inflammation of the adnata returned, universal prostration of strength followed, and the patient expired on the night of the 22d. From the moment the powers of speech were suspended or rather annihilated, the iris lost the property of contracting and dilating; and neither the application of strong light, of objects drawn rapidly across the eyes, or even frictions on the cornea, made the smallest impression, whilst the orbicularis palpebrarum, the Levator palpebræ superioris, and muscles of the globe continued in full activity. There was much inclination to sleep, frequent startings, craunching or grinding of the teeth, constant moaning, and a tendency to strabismus, the axis of the left eye being directed towards the nose; but the pupils were not in the slightest degree dilated in any stage of the disease; there was likewise a paralysis of the whole left side, which was observed for the first time on the 16th. The blood drawn from the arm was one homogeneous mass, without any inflammatory marks of buffy gluten on the surface, although taken from a large orifice. It was discovered during the delirium, that he laboured under virulent gonorrhœa, of which the patient gave not the least previous intimation.

In reflecting on all the circumstances and symptoms attending this case with its fatal termination, I was extremely anxious to examine into the state of the brain, in order to ascertain precisely the seat of the disease; in some measure persuaded however, that death, if not solely owing to compression from an effusion of lymph or serum into the ventricles, it would at least be found a mixed case, that is, combined of congestion and effusion: I therefore requested Mr. Fuge, first Surgeon of the Hospital, to open and examine the contents of the cranium in my presence on the following day, which he very readily complied with, accompanied by his son, a very promising young gentleman and a good anatomist, who, to save his father the trouble, performed the operation with much neatness and science.

On making an horizontal section of the bones of the cranium, and removing the superior portion, to which the dura mater adhered very firmly, the vessels of the brain were found unusually and prodigiously distended with blood, the cerebrum and cerebellum exhibiting the most striking resemblance of a beautiful anatomical preparation, on which the greatest violence and utmost force of injection had been employed. When the dura mater was carefully detached, its internal surface appeared at first as if covered with coagulable lymph from inflammation; but on closer inspection, I considered it only a portion of the fine pellucid fluid condensed after death, which every where exudes from the surface of the pia mater. The minute vessels of the plexus choroides were likewise distended, and the torcular Herophili sinus was full of blood; but to to my great astonishment the ventricles were wholly free from any compressing fluid, the fourth contained a small quantity, probably not more than what is usually found in these cavities when the brain is in a perfectly sound state. It now became manifest that the patient died apoplectic from congestion, or a preternatural accumulation of blood in the vessels of the sensorium, originally depending probably on loss of tone in these vessels.

A knowledge of the characteristic distinctions between universal and local inflammation, is of the greatest importance in the treatment of this and other diseases: By the former is understood general increased vascular tone combined with excess of irritability; by the latter, morbid derangement and excessive irritability in a particular and individual organ. The cure of the first, as already observed, is effected by general means only, while in the second, we are obliged to combine general with topical remedies, as the situation of the affected organ is more or less remote, that is, internal or external, and which in their application often require much penetration and practical knowledge in the physician. The unequal distribution of the circulation destroying the equilibrium, as in local inflammation, is often attended with lesion; and hence the necessity of an early and judicious treatment. The means of lessening local action, merits particular attention, as by its increase or diminution, a more uniform distribution of the sensorial power is effected. The debilitating applications, or means, must be proportioned to the excess of irritable action; if carried beyond this, there will be danger of injuring the organization and vital energy of the part.

It is exceedingly difficult to distinguish the gradation of symptom and nice shades of difference between the local Sthenic Congestion and the Atonic. The chronic state of inflammation is commonly superinduced by a long course of intemperance and low debauchery, as it is a disease frequent among a certain description of seamen and marines; indeed, my experience confines it principally to these classes of people; and the numbers that are affected with a slighter degree of it, is truly surprizing, often terminating in vertigo and incurable head-aches: It affects them more in winter than in summer, owing to the diminished action on the surface, and consequently a greater determination to the internal organs. It chiefly prevails among those constitutionally debilitated by inebriation, and who in their youth had been brought up to mechanic arts which compelled them to sedentary lives. It is also probable, that that it is a frequent complaint in large manufactoring towns, where confinement, bad air, and poverty, predispose the system to such attacks.

From what has been observed above respecting the predisponent causes of this disease, it will be found the most hopeless of all the three species, when once arrived at that stage of it which produces Apoplexy. General bleeding, it is to be feared, would still farther depress the vis vitæ, without relieving the congestion; topical blood-letting only must then be employed, and it will be necessary probably to observe some limitations even to this: Blisters are to be applied to the sutures, rubefacients to different parts of the head, stimulating salts to the nose, the extremities immersed in hot water, and every other means that can be devised to rouse the energy of the brain, and restore the elasticity of its vessels, already weakened if not destroyed by distension. When from previous symptoms, and before congestion is completely formed, the nature of the malady is foreseen, the application of the whole or a part of the above means, will in all probability be sufficient to ward off the impending evil; but to these must afterwards be conjoined the judicious internal use of those stimuli which are known to possess powers to excite the action of the nervous system, and give tone to the lax fibre. This species of Apoplexy often makes a near approach and has characters similar to Paralysis, and is sometimes accompanied by Hemiplegia. I have had under my care, at different times, a strange variety of these kind of complaints, which would be extremely difficult to reduce to nosological order and system, or to describe even their anomalous symptoms with any degree of accuracy and precision.

There are three points which should be impressed as essential to the successful treatment of general and local Sthenic Congestion. 1st. When general bleeding is to be employed, to make choice on every occasion of the jugular veins for the operation. 2d. During that operation, to make compression as far as it may be possible on the carotid arteries, to obstruct or retard the circulation to the brain: I have many times relieved head-ach, fulness and giddiness, to which I have been myself subject, by compressing these arteries with my fingers. And, lastly, never to omit the application of cold frequently renewed to the head.

I venture to offer these facts and observations to the public with great diffidence; but if what is here advanced, with as much brevity as the importance of the subject would would admit, has thrown any additional light on the nature and treatment of Apoplexy, or should have the effect of reconciling and conciliating the contradictory sentiments and differences of others, by pursuing a middle course, it will have fully answered its intended purpose; and should it even fail in these respects, it may at least tend to stir up a spirit of research in others, better qualified, from possessing a greater fund of facts, and greater powers of intellect, to elucidate a point in which so many inconsistent and opposite opinions have been lately given in the Medical and Physical Journal.

*March* 9, 1803.

